# Burden of NCDs in SNNP region, Ethiopia: a retrospective study

**DOI:** 10.1186/s12913-018-3298-0

**Published:** 2018-07-04

**Authors:** Misganu Endriyas, Emebet Mekonnen, Tadele Dana, Kassa Daka, Tebeje Misganaw, Sinafikish Ayele, Mekonnen Shiferaw, Tigist Tessema, Tewodros Getachew

**Affiliations:** 1 SNNPR Health Bureau, Hawassa, Ethiopia; 2 College of Health Science and Medicine, Wolaita Sodo University, Wolaita Sodo, Ethiopia

**Keywords:** Non-communicable diseases, Digestive disorder, Cardiovascular diseases, Diabetes mellitus, SNNPR, Ethiopia

## Abstract

**Background:**

Non-communicable diseases (NCDs) are medical conditions or diseases that are non-transmissible. As NCDs are becoming one of major public health problem, providing local description of diseases and injuries is key to health decision- making and planning processes. So, this study aimed to describe caseload of NCDs in Southern Nations Nationalities and People’s Region, Ethiopia.

**Methods:**

A facility based retrospective study was conducted in February 2015 in SNNPR, Ethiopia. A total of 22,320 records of three years retrieved from 23 health facilities using systematic sampling. Data were entered in to Epi-Info 3.5.3 and descriptive analysis was carried out using SPSS version 20.

**Results:**

From 22,320 records reviewed, 6633 (29.7%) clients visited health facilities due to Non-Communicable Diseases (NCDs). Majority (37.2%) of NCD cases were in productive age groups (20–35 year). Near to half (43%) of NCD cases were from rural and 45.8% were females. Digestive disorder (26.7%), cardiovascular diseases (18.8%) and Diabetes Mellitus (13.1%) were the most prevalent types of NCDs.

**Conclusion:**

Health facilities are burdened with significant proportion of clients with NCDs. Young population accounts large share and NCDs are becoming public health problem of urban and rural area within a health care system that focus on communicable diseases. There is a need to strengthen the health system to work towards NCDs, and investigate risk factors associated with NCDs at individual level.

## Background

Non-communicable diseases (NCDs) are medical conditions or diseases that are non-transmissible [1]. Cardiovascular diseases, cancers, chronic respiratory diseases and diabetes are the four leading types of NCDs [1–3]. Risk factors for NCDs are categorized in to modifiable (behavioral), metabolic and physiological risk factors. Modifiable behavioral risk factors comprise tobacco use, physical inactivity, unhealthy diet and harmful use of alcohol [1]. An important way to reduce NCDs is to focus on lessening above mentioned risk factors, early detection and timely treatment [4].

NCDs are one of the major health problems and in 2015, it was reported that they were responsible for 68% of the world’s deaths [5]. Almost three quarters of all NCD deaths and the majority (82%) of premature deaths occur in low- and middle-income countries [5]. In African nations, NCDs are rising rapidly [4, 6–8] and are projected to exceed communicable, maternal, perinatal, and nutritional diseases as the most common causes of death by 2030 [4]. World Health Organization (WHO) 2014 country profile reported that about 30% of total deaths in Ethiopia were associated with NCDs from which cardiovascular diseases, cancers, chronic respiratory diseases, diabetes, and others NCDs accounted for 9, 6, 3, 1 and 11% respectively. It was also estimated that the probability of dying between ages 30 and 70 years from the four main NCDs was 15% [9].

Despite these facts, these diseases have not been given adequate attention due to the overwhelming burden of infectious diseases [6]. In the context of epidemiologic transition in Ethiopia, a double burden of disease is already emerging with mix of persistent communicable disease and increasing NCDs [10, 11]. However, the burden of NDCs is believed to be under estimated due to lack of reliable data and lack of disease registration system [8, 11].

A reliable description of diseases (health conditions) and their risk factors is an important input to planning and decision- making [12, 13]. But considerable proportion of countries (including Ethiopia and study region) have little usable mortality data and weak surveillance systems [4, 11]. So, this study was carried out to provide local evidence on burden of NCDs (in terms of caseload) to inform decision-makers and improve planning on NCDs in Southern Nations Nationalities and People’s Region (SNNPR), Ethiopia.

## Methods

Facility based retrospective study was conducted in SNNPR Ethiopia in February, 2015. SNNPR is the third largest administrative region in the country and is the most diverse region in terms of language, culture and ethnic background. Administratively the region is divided into 14 zones, 1 city administration and 4 special woredas. Woreda, equivalent to district, is government administrative structure in zone with approximately 100,000 population while special woreda is woreda directly accountable to region, not contained in zone. Based on 2007 census, the region had an estimated population of 18.9 million in 2014 [14]. According to regional health bureau 2013/2014 annual report, there were a total of 21 governmental hospitals, 703 health centers and 3835 health posts [15]. Health posts mainly perform preventive activities at community level while health centers perform both curative and preventive activities leading (on average) 5 satellite health posts in their catchment.

Considering existing logistics [16, 17], a total of 23 health institutions were included. Facilities were from 12 zones, 1 special woreda and 1 city administration and were geographically representative. Since majority of health centers in the region were relatively new (not well equipped to diagnose NCDs), capacity of health facilities to diagnose and treat NCDs was considered as inclusion criteria. Government (hospitals and health centers) and private (hospitals and higher clinics) facilities that diagnose and treat NCDs based on physician and laboratory investigation were included in the study.

Three years (2012–2014) clients’ records were reviewed. Sample size for clients’ card review for single facility per year was calculated by using the single population formula as follows

$$ n\ge {\left[\frac{z_{\raisebox{1ex}{$\alpha $}\!\left/ \!\raisebox{-1ex}{$2$}\right.}}{E}\right]}^2p\left(1-p\right) $$

Where n-sample size, P- proportion of population (prevalence), Z- reliability coefficient and d- margin of error. Using *P* = 30% as WHO 2014 report stated 30% national disease burden were due to NCDs [9], Z = 1.96 reliability coefficient for 95% confidence interval and d = 0.05, sample size for a facility per year was 323. Since we reviewed three years record, 969 records were expected to be reviewed from each facility. Rounding 969 to 1000, final sample size was 23,000.

As malaria was endemic and seasonal in some areas in Ethiopia [18] and in SNNPR, major transmission season (September to December) records were not included as malaria epidemic may influence true burden of cases flow. So, we considered records from January to April.

Because of the challenge to review individual client card from medical record unit (card room) due to its arrangement, mixing, lacking level and even missing in some facilities, we used registers to review caseloads. We collected all registration books from all departments (inpatients, outpatients, maternal and child, specialty, emergencies etc.…) and counted cases in each registration books over defined study periods and then sample size for each year was allocated for registration books based on cases registered on registers. Finally, the allocated sample size was reviewed using systematic random sampling. During review, records with incomplete, missing or un-readable data were replaced with next records to compensate sample size and continued until specified sample size was filled.

We extracted socio-demographic data, final diagnosis and outcome (alive/dead). For multiple health conditions, specialists were consulted to classify main diagnosis. Records with death outcome were collected without confirming death certification as there was no certification system.

Diseases (health conditions) were classified into: Group I (communicable diseases); Group II (non-communicable causes); and Group III (injuries) and Group IV (maternal conditions and nutritional deficiencies). The magnitude of diseases/conditions attributable to each group were computed.

Data was collected by 11 trained data collectors who have bachelor in health officer or nursing using structured and pre-tested checklist. Data entry and cleaning was done using Epi Info version 7 while descriptive analysis was done using IBM SPSS version 20 for windows. Data cleaning was done by going through the variables one by one and cross tabulating to check for any inconsistencies and out-lying values. Errors during data entry were resolved by going back to the original checklist. Frequencies and percentage of different variables were computed and the results were presented using tables and figures.

Ethical clearance was obtained from SNNPR health bureau Ethical Review Committee. Administrative permission was obtained from regional health bureau and zonal health departments. Verbal consent was obtained from heads of health facilities and data handlers after clear explanation of the purpose of the study. No individual identifier data was collected and all information collected was kept confidential.

## Results

### Overview of health facilities and study population

A total of 23 health facilities (15 government hospitals, 1 private hospital, 3 government health centers and 4 private higher clinics) were included in this study. These health facilities were selected from 12 zones, 1 special woreda and 1 city administration indicating facilities were geographically representative to the region. From these facilities, 22,320 records were reviewed.

About half (50.8%) of clients were females. The age of clients ranged from birth to 99 years with mean of 26.5 years (SD 16.9). The age distribution of clients showed a large number of clients were in age group of 20–35 (39.4%) and under 5 years old (11.6%). From total records, 13,203 (59.1%) clients were from urban areas and 8924 (40.3%) were from rural areas while 193 (0.9%) had missing records of residence (Table 1).

**Table 1:** Distribution of sex, residence and age category of clients in SNNPR, 2012–2014

Variable	Category	Frequency	Percent
Sex	Male	10,985	49.2
	Female	11,335	50.8
Residence	Rural	8924	40.0
	Urban	13,203	59.1
	Missing	193	0.9
Age	< 5	2587	11.6
	5–9	1337	6.0
	10–14	1164	5.2
	15–19	2009	9.0
	20–24	3330	14.9
	25–29	3270	14.7
	30–34	2179	9.8
	35–39	1719	7.7
	40–44	1342	6.0
	45–49	992	4.4
	50–54	802	3.6
	55–59	398	1.8
	60–64	473	2.1
	65–69	247	1.1
	70–74	241	1.1
	75–79	82	0.4
	80+	148	0.7
	Total	22,320	100.0

### Cause of visit

Of 22,320 records, leading cause of visit was infectious diseases which accounted 11,847 (53.1%), followed by NCDs 6633 (29.7%). However, when cases of NCDs added with injuries, figure exceeded one-third (37.1%) (Table 2).

**Table 2:** Distribution of diseases/conditions among clients, SNNPR, 2012–2014

Disease/illness/health condition	Frequency	Percentage
NCDs	6633	29.7
Infectious	11,847	53.1
Maternal and nutrition	2184	9.8
Injuries	1656	7.4
Total	22,320	100.0

### Description of NCDs

Large number (37.2%) of clients with NCDs were in age range of 20 and 34 years old and more than half (57.0%) were living in urban areas (Table 3). Female to male sex ratio showed slight difference (0.86), male contributing 54.2% of total NCD cases.

**Table 3:** Distribution of NCDs by residence, sex and age category, SNNPR, 2012–2014

Variable	Category	Number	Column %
Residence	Urban	3779	57.0
	Rural	2854	43.0
Sex	Male	3594	54.2
	Female	3039	45.8
Age	< 5	176	2.7
	5–9	182	2.7
	10–14	241	3.6
	15–19	562	8.5
	20–24	840	12.7
	25–29	915	13.8
	30–34	711	10.7
	35–39	644	9.7
	40–44	576	8.7
	45–49	486	7.3
	50–54	405	6.1
	55–59	227	3.4
	60–64	270	4.1
	65–69	124	1.9
	70–74	137	2.1
	75–79	47	0.7
	80+	90	1.4
Total		6633	100.0

Of 6633 NCDs reviewed, digestive disorders (26.8%), cardiovascular diseases (18.8%) and diabetes mellitus (13.1%) were top three NCDs among others (Table 4).

**Table 4:** Types of NCDs in selected health facilities, SNNPR, 2012–2014

Types of Non-communicable disease	Number	Percent
Diabetes Mellitus	870	13.1
Epilepsy	418	6.3
Cancer	316	4.8
CVD	1246	18.8
Chronic Respiratory Disease	389	5.9
Digestive Disorder	1775	26.8
Mental Health Problem	631	9.5
BPH	164	2.5
Eye Disease	186	2.8
Autoimmune Diseases	180	2.7
Neurological disorder	51	0.8
Renal Disorder	126	1.9
Other NCDs	281	4.2
Total	6633	100.0

Majority of under-five clients with NCDs, 150 (85.3%), were diagnosed in hospitals and only 8 (4.5%) were diagnosed in health centers from which half were chronic respiratory diseases.

**Fig. 1:**
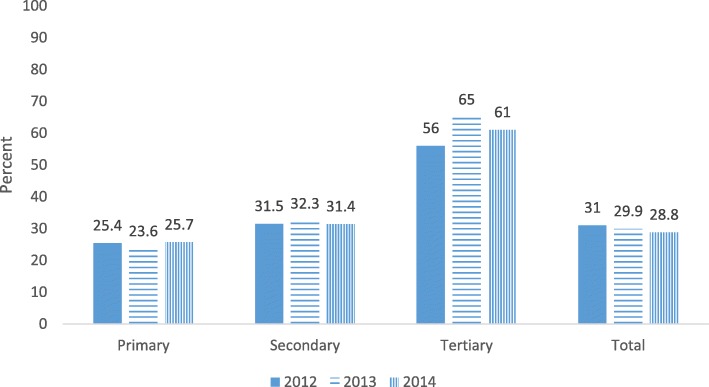
Percent of NCDs in primary, secondary and tertiary level of health facilities, SNNPR, 2012–2014

The proportion of NCDs from total clients was compared over facility levels and differences were noted among primary, secondary and tertiary levels. Percentage of NCDs in tertiary hospital was twice that of secondary (general) hospitals. About one fourth of clients (24.8%) in primary health facilities and more than half of clients (59.7%) in tertiary hospital had at least one of NCDs. The trend of NCDs in all levels changed slightly over three years (Fig. 1). In addition, proportion of clients with NCDs were higher in private facilities (1665 (34.9%)) as compared to government owned facilities (4968 (23.8%)).

### Outcome

From total records, 72 deaths registered from which more than half, 42 (58.3%), were females. Moreover, 32 (44.4%) and 25 (34.7%) clients who were died came to health facilities due to infectious and NCDs respectively (Table 5). A high number of deaths were observed among under five children and 25–29 years old young group which accounted 21 (29.2%) and 12 (16.7%) respectively. More than half 38 (52.8%) of all deaths registered came from rural areas.

**Table 5:** Causes of deaths in selected health facilities, SNNPR, 2012–2014

Causes of death	Frequency	Percent	Cumulative percent
CVD	9	12.5	12.5
Malaria	8	11.1	23.6
Digestive Disorder	7	9.7	41.7
Pneumonia	6	8.3	31.9
Malnutrition	4	5.6	47.2
HIV/AIDS	3	4.2	51.4
Meningitis	3	4.2	55.6
Epilepsy	3	4.2	59.7
Fighting	3	4.2	63.9
Typhoid fever	2	2.8	66.7
TB	2	2.8	69.4
AFI	2	2.8	72.2
Cancer	2	2.8	75.0
Others	18^a^	25.0	100.0

^a^- all 18 other causes of death were of different categories

## Discussion

This study was proposed to show the magnitude of NCDs in health facilities in SNNPR over three years and covered 23 health facilities of different types (primary, secondary and tertiary levels, and private and public owned facilities).

The overall magnitude of NCDs among reviewed 22,320 patient cards was 6633 (29.7%) and was in line with WHO country profile report of 2014 [9] which reported 30% of burden comes from NCDs. It was also comparable with findings of study conducted in Nepal using facility based data [19] that reported overall prevalence of NCDS to be 31%.

Evidences show that there is an increasing burden due to NCDs [5, 6, 8, 14, 20–28]. Different community based disease specific surveys on NCDs like diabetes and hypertension in Ethiopia also concluded that the prevalence of these diseases is high [28–41]. In this study, the overall prevalence of NCDs is also high and is alarming in country where infectious diseases are among major cause of morbidity and morbidity. Even though communicable diseases were the leading causes of morbidity in this study too, the magnitude of NCDs is also approaching to infectious diseases, being the next major causes of morbidity. While the prevalence of NCDs is high, it changed slightly over three years being 30.4, 28.8 and 28.7% for years 2012, 2013 and 2014 respectively. The slight change might be due to the short period of time included to see trend because of limited availability of data.

Even though the prevalence of NCDs is increasing, chronic NCDs in developing countries (including Ethiopia) have got little attention due to competing challenges [6, 20, 42, 43]. In addition, lack of a national strategy for prevention and control of NCDs has been hampering coordination of activities and services to minimize potential risk factors and manage diseases [43].

Above studies conducted in Ethiopia [28–41] associated NCDs with different socio-demographic and socio-economic factors, more specifically with low awareness and poor practice and recommended policy and strategic actions that could minimize risk factors. Evidences show that many of NCDs related health-care interventions are cost effective if provided early, compared to costly procedures at advanced stages of diseases [4, 44], but many of acute interventions required to address NCDs are best provided at referral (hospital) level and require health workers with advanced training [4, 44, 45]. One of the key steps for prevention and control of NDCs is preparing national strategies to guide the control and prevention activities [45]. However, national guidelines (strategies) and appropriate care (technologies and medicines) for people with NCDs is lacking in low-income country, Ethiopia [43].

Another thing that should be taken in to account is the distribution of NCDs was alarmingly comparable between urban and rural areas and attacking reproductive age group. Studies in other country [46] and in Ethiopia [37] also reported that NCDs are becoming problem of both urban and rural communities. So, health systems need to be further strengthened to deliver an effective, realistic and affordable package of interventions and services for people with NCDs.

This study was first comprehensive study on prevalence of NCDs in the region using relatively big samples and provides useful background data to stakeholders working on NCDs and researchers. As facility based retrospective study and due to its limited methods, this study has the following limitations. First, due to lack of organized data in regionally representative health facilities, trend presented was of short period. Second, the magnitude presented here was caseload of NCDs and didn’t control recount of cases by revisit and referral. In addition, even though analysis of records may help to understand what went wrong (medical errors) and what measures could be taken to minimize such errors in the future [47], we used final diagnosis and didn’t review symptoms, signs and classification due to limited logistics (money, time and human power). Finally, the proportion shown here may not represent true prevalence in community as it doesn’t represent people who are not accessing healthcare (and died before accessing) due to different factors.

## Conclusion

Health facilities are burdened with significant proportion of clients with NCDs. Young population accounts large share and NCDs are becoming public health problem of urban and rural area within a health care system that focus on communicable diseases. There is a need to strengthen the health system to work towards NCDs, and investigate risk factors associated with NCDs at individual level.

## Abbreviations

BPH: Benign Prostatic Hyperplasia
CVD: Cardiovascular Disease
IBM: International Business Machines Corporation
NCDs: Non-Communicable Diseases
SNNPR: Southern Nations Nationalities and People’s Region
SPSS: Statistical Package for the Social Sciences
